# Supplementary material for: Are carbon emissions trading and green financial instruments synergistic? -Comprehensive quantitative research based on content analysis
Source: PLoS One. 2024 Mar 7;19(3):e0298601. doi: 10.1371/journal.pone.0298601 (PMC10919601; doi:10.1371/journal.pone.0298601)
Supplement: S1 Data — (ZIP) [file pone.0298601.s001.zip › Data/地级市Z_F工作报告.zip!/░▓╗╒/┬φ░░╔╜╩╨/└┤╫╘╣1⁄2╓┌║┼í╛┬φ┐╦╩2╛▌═°í┐_2013.docx]

# **政府工作报告（2013）**

【字体大小：[大](http://zwgk.mas.gov.cn/openness/detail/content/javascript:doZoom(18)) [中](http://zwgk.mas.gov.cn/openness/detail/content/javascript:doZoom(15)) [小](http://zwgk.mas.gov.cn/openness/detail/content/javascript:doZoom(14))】

政府工作报告

——2013年1月6日在马鞍山市第十五届

人民代表大会第一次会议上

市 长   张晓麟

各位代表：

现在，我代表市人民政府，向大会作工作报告，请予审议，并请市政协委员和其他列席人员提出意见。

一、去年工作回顾和过去五年发展成就

刚刚过去的2012年，是本届政府任期届满之年，也是全市上下攻坚克难负重前行、各项工作全面推进的一年。面对宏观环境复杂多变等不利因素，在省委、省政府和市委的坚强领导下，市政府团结带领全市人民，深入贯彻落实科学发展观，以迎接党的十八大胜利召开为强大动力，围绕市第八次党代会提出的抓好“三件大事”、建设“四个中心”，做规划、打基础，拉框架、建平台，稳增长、促发展，惠民生、构和谐，较好地完成了市十四届人大六次会议确定的各项目标任务，在建设小康征程上迈出了坚实步伐。

预计全市实现地区生产总值1200亿元，增长12%。固定资产投资突破1000亿元，达1200亿元，增长26%；财政收入突破200亿元，达210.6亿元，增长13%；规模以上工业增加值突破500亿元，达530亿元，增长14%；城市居民人均可支配收入突破3万元，达30900元，增长13.1%；农民人均纯收入突破1万元，达11200元，增长17.8%，主要指标实现了“五大突破”。博望区成立，慈湖高新区升级为国家级高新区，郑蒲港新区挂牌并获批为省级现代产业园区，承接产业转移集中示范园区获准设立；我市成为全国创业先进城市，再次入选中国外贸百强城市，顺利通过国家环保模范城市复核，全国城市文明程度指数测评居84个参评地级市第2，城市居民幸福指数居全省之首，科学发展指数居全省第2，全面小康社会实现程度达97.1%。

（一）经济平稳较快发展

“项目推进年”活动成效明显。新开工建设项目1750个，同比增加310个；400个市重点项目累计完成投资630亿元。马钢高速车轮用钢等72个项目建成投产，数字硅谷产业园等127个项目开工建设，康佳绿色科技产业园等62个10亿元以上项目签约，马钢（合肥）环保搬迁项目获批，过江通道等一批重大基础设施项目谋划取得积极进展，万达广场创全市项目推进速度最快新记录。开展工矿废弃地复垦调整利用试点，完成土地整治19万亩，获批建设用地3.96万亩，供地4.5万亩，闲置土地处置率达96%，国土“四模”创建工作得到国土资源部、省政府肯定。推进征迁拆迁，完善政策措施，实行项目包干征收，强化评估监管，完成拆迁面积515万平方米。完善投融资体制，创新融资方式，政府融资规模达117亿元，城投集团融资规模突破50亿元，多项指标居全国同类城市前列。民生银行马鞍山分行开业运营，中信银行获准筹建；金融机构本外币贷款余额902亿元，新增100亿元；各类社会融资超400亿元。

三次产业平稳增长。新增规模以上工业企业104户，总数665户。制定落实服务企业22条具体措施，举办百强企业发布会。马钢面对前所未有的困难，加强内部改革管理，努力降本增效。农业实现增加值70亿元，粮食产量再创历史新高，30个现代农业示范园完成投资20亿元，市级以上龙头企业实现营销收入58亿元，农产品加工产值增长20%。服务业实现增加值326亿元，增长11.2%；社会消费品零售总额262亿元，增长16.1%。旅游业总收入100亿元，增长23%，在建五星级酒店5家；房地产业健康发展，新建商品房销售面积279万平方米，增长43%，住房公积金归集和使用规范有序；举办首届海峡两岸电子信息博览会等各类展会62场，地方名特优产品培育销售工作扎实有效，成为国家农产品现代流通综合试点城市。建筑业实现总产值312亿元，增长30%。

（二）多点支撑格局形成

县区实力显著增强。县区财政收入占全市的56.4%，固定资产投资占全市的68.2%。含山县固定资产投资突破百亿元，增幅超过50%，连续两年被评为皖江示范区建设先进县。和县地区生产总值、固定资产投资双双超百亿，名列全省县域分类考核二类县第2名。当涂县地区生产总值增长19.5%，固定资产投资突破300亿元，再度跻身中国中小城市科学发展百强县行列。花山区现代商业加快发展，软件园开园、3户上市公司进驻，濮塘一批重点旅游项目开工。雨山区国家级循环经济再制造重点示范项目开工建设，东部电子新材料产业园初具规模，亿丰环球购物中心即将建成。博望区新城区规划和基础设施建设全面启动，国家火炬剪折机床及刃模具特色产业基地获批。

园区新区蓬勃发展。市经开区引进亿元以上项目22个，其中超10亿元项目4个；66个项目竣工投产，成为全省首个省级电子信息产业园。慈湖高新区扩区托管、资源整合平稳实施。郑蒲港新区创新体制机制，产业区和新城区建设同步推进，皖江最大等级的深水码头开工建设。示范园区成为省现代服务业集聚区，协会招商、园中园招商成效明显。滨江新区沿江大道建成通车，安置房建设快速推进。秀山新区建设全面提速，东部环路竣工通车，秀山湖主体工程完工。6个园区、新区完成固定资产投资占全市的29.4%。

（三）产业转型步伐加快

产业层次得到提升。国家铁基新材料高新技术产业化基地获准设立，装备制造等优势主导产业加快发展。谋划“一基地四园区”（港口物流基地和电子信息、汽车及零部件、绿色建筑装饰材料、钢铁生产交易产业园），规划、宣传、招商同步推进。71个重点技改项目竣工投产，战略性新兴产业产值增长43%。新认定国家火炬重点高新技术企业5户、高新技术企业36户、高新技术产品170个，4家省级工程技术研究中心、2家省级重点实验室，1个院士工作站、4个博士后科研工作站获准设立。产学研合作进一步深化。授权专利突破2000件，增长45%。国家钢铁及制品质检中心通过验收，“马钢”（合金钢、钢板）、“山鹰”、“三联”3件商标荣获中国驰名商标，“奥盛”等26件商标成为省著名商标。

节能减排扎实推进。开展节能减排攻坚行动，实施9个重点节能项目，加快淘汰落后产能，6座计划关闭小高炉全部关停；东部污水处理厂竣工投入运营，新建污水管网39.8公里，污泥处置中心、有机生物处置中心完工，4台燃煤发电机组脱硝设施建成运行，机动车排气污染防治管理成效明显，节能降耗、主要污染物排放量可望控制在省下达指标以内。

（四）改革开放不断深入

重点领域改革稳步推进。推动工程建设领域和土地房屋征收管理体制改革向县区延伸覆盖，招标采购市场行为进一步规范。完成“营改增”试点第一阶段工作。积极探索事业单位人事分配制度改革，事业单位分类改革稳步推进。全面启动县级公立医院综合改革，实现药物零差率销售，市级公立医院改革试点不断深入。成为全国国有文艺院团体制改革工作“突出贡献地区”。服务业统计工作扎实推进，企业一套表改革工作受到国家表彰。非公经济加快发展，新发展民营企业2822户、个体工商户1.2万户。

对外开放水平不断提高。整合招商资源，设立6个驻外招商联络处，成立32个产业招商小组、6个招商咨询小组，实行信息共享，完善考核办法。产业招商、专业招商、协会招商、会展招商扎实开展，实际到位内资1260亿元，增长51.8%；到位外资13亿美元，增长30.4%；与21个国家级协会学会达成战略合作。对台招商取得重要成果，正崴科技园等7个台资项目成功引进。完成外贸进出口35.3亿美元，外经营业额突破3亿美元。拓宽人才引进渠道，在清华大学举办人才招聘会。深化宁马一体化合作，宿马现代产业园建设快速推进。

（五）城乡承载功能提升

基础设施进一步完善。实施城乡基础设施项目475个，完成投资420亿元。长江公路大桥全面进入上部结构施工，城际铁路站前广场地下枢纽工程主体封顶。东环路高速化改造、105省道改建工程竣工通车，314省道升级改造主体完工，马巢高速、205国道升级改造扎实推进，206、226省道拓宽改造启动实施。新建城区主干道路38.3公里，升级改造县乡公路74.4公里。副城区、中心镇和农村新社区建设完成投资80亿元。24个电网建设项目快速推进，供电保障能力提升。

城乡面貌明显改观。启动慈湖河中游综合整治，加快城市出入口环境整治步伐，实施南湖公园景观提升工程，建成4座街头游园。完善城市管理体制，启动数字化城管平台建设，开展“三车”（渣土车、散装运料车、预拌混凝土运输车）治理。完成57个老旧小区整治三年计划。实施滁河防洪治理工程，农田水利基本建设力度加大。美好乡村建设得到省委、省政府肯定，50个示范村加快建设。完善村庄规划编制，实施53个村庄整治，建成10个乡镇压缩式垃圾中转站，改造农村危房6544户。

（六）社会建设和管理得到加强

民生工程深入推进。实施40项民生工程，投入资金30.3亿元，增长23%。新增城镇就业4.5万人，期末城镇登记失业率2.83%。人力资源服务产业园开园。医保付费方式改革全国试点扎实推进，成为全国第四批城乡居民社会养老保险试点城市。城乡低保、农村五保实现应保尽保，市社会福利中心投入使用，惠残民生工程顺利实施。新开工安置房2.8万套，竣工2万套；新开工保障性安居工程1.9万套，竣工1万套。物价水平总体稳定。公交优先发展战略深入实施。

社会事业全面进步。国家公共文化服务体系示范区建设扎实推进，举办李白诗歌节、凌家滩文化论坛，群众文化活动丰富多彩，首家民办博物馆建成开馆。学前教育三年行动计划深入实施，进城务工人员子女享受同等义务教育，学校少年宫建设实现城乡全覆盖，中职园建成使用。基层卫生服务体系不断健全，新农合制度进一步完善。市体育会展中心加快建设，全民健身活动广泛开展。低生育水平持续稳定，“阳光计生”行动获国家表彰。广播电视、新闻出版、文学艺术、哲学社会科学进一步繁荣。档案、地方志工作取得新成绩。外事、侨务、港澳台，地震、气象、防灾减灾工作得到加强。青少年、妇女儿童、老龄工作成效明显，市关工委、老年大学工作成绩显著，青年志愿服务活动广泛开展，红十字工作受到国家表彰。

社会管理水平提升。社区网格化管理实现中心城区全覆盖，农村社区服务设施规范化建设加快推进。领导干部开门接访、带案下访，健全完善三级平台，推行联合接访和信访代理制，信访秩序规范有序。加强社会治安综合治理，开展“重点工程保平安”活动，治安防控体系建设扎实推进，人民群众安全感和满意度测评得分居全省前列。实施“打非治违”专项行动，安全生产形势保持稳定。建立司法行政工作新模式，“六五”普法深入开展。应急管理体制机制进一步完善。产品质量和食品药品安全监管力度加大。民族团结、宗教和睦局面进一步巩固。国防教育、国防动员、人民防空、双拥优抚工作得到加强。驻马部队和广大民兵预备役人员在促进社会稳定、支持地方建设等方面作出了重要贡献。

切实加强政府自身建设，开展“保持党的纯洁性、迎接党的十八大”主题教育实践活动，提高推动科学发展的能力和水平。认真执行市人大及其常委会决议、决定，向市人大常委会报告工作和向市政协通报情况，主动接受市人大的法律监督、工作监督和市政协的民主监督，认真办理人大议案、建议批评意见和政协提案，注意倾听和吸纳各民主党派、工商联、无党派和社会各界人士的意见建议。严格行政决策程序，发挥行政复议化解行政争议主渠道作用。政府信息公开工作规范推进，政务微博开通。科学制定目标管理考核体系，加大督办查办力度，推动工作落实。取消行政审批11项，调整23项，推进并联审批、网上预审、告知承诺制，审批时限进一步缩短。强化重点领域、关键环节的行政监察和审计监督，廉政风险防控工作扎实开展，严格控制“三公”经费支出，政风建设取得新成效。

回顾总结去年工作，需要特别报告的是，规模以上工业增加值尽管实现了14%的增长目标，但没有达到“力争突破600亿元”的目标，主要原因是受外部宏观环境影响，加之自身正处在转型期，工业经济运行困难所致；新增3户上市公司目标未能实现，客观原因是国家收紧拟上市公司发行审核尺度，全省仅1户企业发行上市；慈湖河综合整治工程进展不快，主要是由于环境质量要求大幅提高，需要对原先确定的整治方案、标准、工期进行调整，以将其建设成为集防洪、生态、景观为一体的综合工程，影响了工程的实施进度。

各位代表！2012年是本届政府的收官之年，回顾过去五年的发展历程，我们遇到了前所未有的困难和压力，大家付出了极大的努力和艰辛，取得了辉煌的业绩！

这五年，是综合实力显著提升的五年。地区生产总值迈上千亿台阶，年均增长13.3%；人均生产总值8600美元，是五年前的2.5倍；财政收入翻了一番多，年均增长16.6%；固定资产投资累计4189亿元，是上一个五年的3.7倍，年均增长30.1%；社会消费品零售总额是五年前的2.3倍，年均增长18%；净增销售收入超亿元企业163户，超10亿元企业16户；新增上市公司4户，全市企业总数突破2万户。

这五年，是经济转型取得突破的五年。二产税收占财政收入比重由62.3%下降到30.4%，三产税收由27.8%上升到45.5%；市本级财政占比由66.8%下降到31%，县区、园区、新区财政由33.2%上升到69%；制造业中的钢铁投资占比由39.9%下降到17.2%，装备制造业投资由22.4%上升到31.8%。连续多次被评为全国科技进步先进市，跻身国家知识产权试点市行列，3户企业成为国家创新试点企业。

这五年，是发展空间全面拓展的五年。含山、和县划入马鞍山，市域面积扩大1.4倍；新成立博望区、花山区，主城区面积扩大1倍，两次行政区划调整成为马鞍山发展史上的里程碑。国家级经开区、国家级高新区先后获批，4个省级开发区、2个省级高新区获准设立，多区联动、多点支撑格局形成。完成城乡建设投资1150亿元，是上一个五年的5倍，城市建成区面积由72平方公里扩大到86平方公里，城镇化率达61.2%。完成拆迁面积1252万平方米，拆除城乡各类违法建设169万平方米。长江公路大桥今年将竣工通车，宁安城际铁路明年有望投入运营，连接城乡、贯通内外的快速交通网正在形成，拥江发展的大城市框架初步拉开。

这五年，是发展动力不断增强的五年。投融资、工程建设、招标采购、国有资产管理、行政管理体制改革力度加大，不断深化完善。医药卫生体制改革、全国公立医院改革试点工作扎实推进，义务教育阶段学校移交区级管理，文化体制改革完成。城乡一体化综合配套改革深入推进。新增银行业金融机构9家、小额贷款公司25家、其他非银行业金融机构74家，金融产品和服务创新成效明显。累计实际利用内资3209亿元、外资43.5亿美元，实现外贸进出口总额158亿美元。成为长三角经济协调会成员城市，口岸正式对外籍船舶开放，马鞍山港成为首批与台湾直航港口之一。

这五年，是人民生活明显改善的五年。累计投入资金105.9亿元，实施66项民生工程。城镇职工养老、失业、医疗保险参保率达到98%以上。城乡教育、卫生等公共服务均等化水平不断提高，成为首批国家公共文化服务体系示范区创建城市，在全省率先实现体育健身工程全覆盖。荣获“全国文明城市”、“全国绿化模范城市”、“全国双拥模范城市”称号。城市居民人均可支配收入是五年前的1.9倍，年均增长13.9%；农民人均纯收入是五年前的2.1倍，年均增长16.5%。

各位代表！奋斗充满艰辛，拼搏铸就辉煌。这些成绩的取得，是省委、省政府和市委正确领导的结果，是全市人民团结奋斗、开拓进取的结果。在此，我代表市人民政府，向辛勤工作在全市各条战线上的广大干部群众，向市人大代表、市政协委员，向各民主党派、工商联、人民团体和社会各界人士，向参与、支持马鞍山建设发展的中央和省驻马单位、解放军和武警官兵，境内外投资者、海内外朋友，表示衷心感谢！

我们也清醒地认识到，当前我市经济社会发展中还存在一些问题，主要是：受外部环境的影响，部分企业生产经营比较困难，新的增长点不够多，经济发展还处在负重爬坡阶段；战略性新兴产业和现代服务业发展需要加大力度，资源环境制约日益凸显，节能减排形势依然严峻；中心城市辐射带动和综合服务功能尚显不足，城乡区域发展不够平衡，农村基础设施还相对薄弱；社会建设、社会管理有待加强和创新，影响社会和谐稳定的矛盾仍然较多；少数干部市场意识、忧患意识不够强，敢于负责、敢于担当的勇气还显得不足，效能不高、节奏不快、落实不力的现象还不同程度存在。对此，我们将高度重视，努力加以解决。

二、今后五年的奋斗目标和主要任务

今后五年，是我市加快转变经济发展方式、推动一江两岸协调发展、建成更高水平小康社会的关键时期。党的十八大胜利召开，为马鞍山改革开放和现代化建设指明了前进的方向，极大增强了我们发展的信心。综合分析未来形势，一方面，经济全球化深入发展，我国发展长期向好的趋势没有改变；工业化、信息化、城镇化、农业现代化深入推进，国内市场开发空间更加广阔；我市良好的生态环境和区位、产业、资源优势进一步凸显，皖江示范区先导区、国家主体功能区规划重点开发建设区域、省城镇体系规划“双核”之一中心城市、皖南国际文化旅游示范区等多重利好政策叠加，我市迎来了一个大有可为的黄金机遇期。另一方面，国际环境不确定不稳定因素增多，世界经济复苏将是一个缓慢而复杂的过程；国内发展中不平衡不协调不可持续问题依然突出，发展进程中经济增长的制约条件增加；区域竞争更加激烈，转变发展方式任重道远。我们要科学把握发展规律，主动适应环境变化，不断开创转型发展、加快发展、和谐发展新局面。

做好今后五年的工作，我们要以邓小平理论、“三个代表”重要思想、科学发展观为指导，深入贯彻落实党的十八大和市第八次党代会精神，着力推动思想解放、改革创新，着力促进经济持续健康较快发展，着力加快转变经济发展方式，着力推动一江两岸协调发展，着力保障和改善民生，把马鞍山建设成为全省经济发展的核心增长极，建成更高水平小康社会，为在全省率先实现现代化、加快建设区域中心城市奠定坚实基础。

今后五年的奋斗目标是：全面达小康，总量争三强，人均居前列。2013年，确保如期实现市第八次党代会提出的目标，全面建成小康社会；2017年，实现地区生产总值、城乡居民人均收入比2012年翻一番，主要经济指标总量力争全面进入全省三强，人均主要经济指标继续保持全省前列，达到长三角中等城市水平，建成更高水平小康社会。

今后五年政府工作的主要任务是：

——努力建设具有较强集聚力的区域中心城市。抢抓大桥、高铁通车带来的新机遇，打造江海联运枢纽中心，规划新的过江通道，形成区域性综合交通枢纽，全方位促进客流、物流、商流、资金流、信息流的流通融入，加快各种生产要素的优化集成。按照“一江两岸、组团式、网格化”城市发展格局，打造花山、雨山核心城区和博望、姑孰、年陡、历阳、郑蒲港、环峰清溪6大城市组团，强化各组团内部功能，构建市域快捷畅达交通网。加快新型城镇化进程，促进小城镇合理分工、功能互补、集约发展。着力提升人口集聚能力，城区人口超100万人，建成区面积超100平方公里，城镇化率达到70%。加快发展高端装备制造、电子信息等优势产业，拉长产业链，提高集聚度。大力发展现代商业、休闲旅游、教育等现代服务业，努力建设集商贸、运动健康休闲、高等教育和职业教育为一体的区域性现代服务中心。

——加速构建富有特色、更具竞争力的现代产业体系。坚持工业强市，推动工业化和信息化、战略性新兴产业和传统优势产业融合发展。规模以上工业企业超1200户，规模以上工业增加值超1300亿元。加快建设重型工业制造中心，支持马钢转型升级，铁基新材料高技术产业产值超过3000亿元，战略性新兴产业产值超过1000亿元，培育形成一批百亿级新兴产业集群。坚持创新驱动，全社会研发投入占地区生产总值比重提高到2.6%，高新技术产业产值占规模以上工业总产值比重提高到40%。坚持工业与服务业融合发展，打造生产性服务业重点集聚区。抢抓皖南国际文化旅游示范区建设机遇，加快培育文化、旅游支柱产业。大力发展现代农业，建设绿色食品供应中心，保障粮食安全和重要农产品有效供给。

——着力激发经济社会发展的体制活力和内生动力。推进新一轮综合改革，进一步处理好政府与市场、社会的关系，进一步明晰市与县区的权责关系，在深化行政管理、经济管理、社会管理、城市管理等重点领域、关键环节改革上取得新突破。实行更加积极主动的开放战略，拓展开放的新领域、新空间，推动开放朝着优化结构、提高效益方向转变，五年实际利用内外资翻一番。把招才引智和招商引资结合起来，吸引各类高层次人才，不断优化人才结构。坚持出口与进口并重，加快形成出口竞争新优势，进一步扩大对外投资合作。

——切实把生态文明建设融入经济社会发展全过程。坚持绿色发展、循环发展、低碳发展，努力形成与生态文明相适应的经济结构、社会结构和发展方式。持续推进国土“四模”创建，建成全国节约集约用地模范城市，努力实现“保护零违法、保障零等待”的目标。落实节能减排目标任务，强化水资源节约保护，加强农田水利基础设施建设，完成省“千万亩森林增长工程”建设任务。努力使水环境质量明显好转，大气环境明显改善，生态竞争力进一步提升，建设山清水秀、天蓝地绿的美丽家园。

——让发展成果更多更公平惠及全市人民。优先发展教育，建设更加均衡的基础教育、更具特色的职业教育和更高质量的高等教育。实施就业优先战略和更加积极的就业政策，大力发展非公经济，推动全民创业，促进创业带动就业。实施城乡居民收入倍增计划，提高低收入者收入水平。着力构筑保障基本民生的安全网，实现应保尽保。坚持尽力而为、量力而行，在发展中逐步提高保障水平。加强城乡医疗卫生制度建设，切实提高公共卫生和基本医疗服务水平。加强保障性住房建设和管理，加快旧城改造步伐。引导广大群众树立通过勤劳致富改善生活的理念，促进形成良好舆论氛围和社会预期。经过全市上下的共同努力，向着学有优教、劳有多得、病有良医、老有善养、住有宜居的更高目标迈进，让全市人民过上更加美好生活。

三、2013年主要工作

今年是全面贯彻落实党的十八大精神的第一年，也是新一届政府的开局之年。中央提出继续把握好“稳中求进”的工作总基调，以提高经济增长质量和效益为中心，继续实施积极的财政政策和稳健的货币政策，促进经济持续健康发展。我们要认真贯彻落实中央、全省经济工作会议和市委八届七次全会精神，坚持稳中求进、好中求快，突出调结构、转方式，开展“项目提速年”活动，以项目提速促进工作提效，全面建成小康社会，实现本届政府的扎实开局。

综合考虑，今年经济社会发展的主要预期目标为：地区生产总值增长12%，财政收入与地区生产总值同步增长，全社会固定资产投资增长20%，社会消费品零售总额增长15%，城市居民人均可支配收入增长13%，农民人均纯收入增长14%，城镇登记失业率控制在4%以内，人口自然增长率、单位生产总值能耗、主要污染物排放量控制在省下达指标以内。

为实现上述目标，我们将认真做好以下重点工作：

（一）着力推动项目建设提速

持续扩大有效投入。牢牢把握扩大内需这一发展的战略基点，发挥投资对经济增长最直接、最有效的拉动作用。全年实施重点项目360个，完成投资750亿元，其中续建马钢（合肥）环保搬迁等160个亿元以上项目，新开工正崴科技园等120个亿元以上项目，推进80个10亿元以上项目前期工作，建成华菱大功率发动机等62个项目。围绕国家投资政策导向，结合我市实际，在重大基础设施、战略性新兴产业、先进制造业、现代服务业等重点领域谋划储备一批牵动性强的大项目、好项目，充实完善重点项目库，争取更多项目落地。

巩固提高要素保障水平。进一步理清思路，合理排序，项目开工前落实好土地、拆迁、资金等保障要素和水电气、消防、通信等配套设施，为项目快速推进创造条件。推进工矿废弃地复垦调整利用试点，实施“万顷良田”土地整治工程，完成土地整治20万亩，获批建设用地3万亩。规范土地和房屋征收，推行成片征收模式，统筹谋划、提前规划建设安置房，完成征迁拆迁面积600万平方米。抓住国家适当扩大社会融资总规模、切实降低实体经济发展融资成本的机遇，进一步提高投融资管理水平，全市融资160亿元以上，城投集团融资50亿元以上。做大做强各级融资平台，市级平台净资产突破300亿元。推动“金融下乡”，提升县区融资能力。

促进各项工作效能提升。落实个人领衔负责制，强化项目协调调度。简化审批程序，缩短审批时限，加强服务标准化建设。提升招投标管理水平，建立优质大型企业库，推行项目打捆招标，推动公共资源交易市场建设。加强过程管控，严格合同约定，提高违约成本。建立项目开工、竣工时间公示、公开制度，广泛接受社会监督。强化项目巡查、督查、考核，定期通报、点评项目推进工作，严格责任追究，确保“项目提速年”活动取得实效。

（二）着力提高经济发展质量和效益

增强创新驱动发展新动力。提升国家级慈湖高新区品牌效应，推动“一区多园”整体升级、跨越发展，加快省级博望高新区建设。强化企业创新主体地位，支持企业牵头实施产业目标明确的重大科技项目。新认定高新技术企业40户、高新技术产品200个以上。深化做实与知名高校、科研院所、学会协会产学研一体化合作。抓好国家知识产权示范市培育和质量强市工作，推动国家级刃模具暨机床质量监督检验中心建设。加大中国驰名商标、省著名商标培育力度，专利授权量增长20%以上。

促进产业结构转型升级。新增规模以上工业企业100户以上，规模以上工业增加值增长14%。以高端装备制造、汽车等为重点，大力发展铁基新材料产业。支持马钢建设发展，加大体制管理创新力度，提升企业核心竞争力。康佳绿色科技产业园等项目开工建设，推动战略性新兴产业加速崛起。进一步优化民营经济发展环境，促进科技型、创新型中小微企业加快发展。着力提升服务企业的针对性和有效性，强化企业靠产品品质赢得市场、靠管理内功赢得竞争的理念，培养更多具有战略眼光、开拓精神、创新能力和社会责任感的优秀企业管理者。

加快发展现代服务业。建设“一基地四园区”，尽快形成产业集聚效应。举办各类展会60场以上，提高办展水平，打造会展品牌，发展会展经济。加大地产品销售力度，提升地方名特优产品知名度。突出运动、健康、休闲旅游特色，加大旅游资源整合和景区景点开发力度。加快形成主城区核心商圈，完善农村商品流通网络，挖掘城乡消费潜力。促进房地产业健康发展。大力发展电子商务，加快发展金融保险、工业设计、文化创意等生产性服务业和面向民生的家政、养老等生活性服务业，形成服务业新的增长点。

（三）着力推动城乡发展一体化

加快发展现代农业。加强现代农业示范区建设，推进当涂现代农业示范区争创国家级。加快农业产业化步伐，培育壮大龙头企业，新增亿元以上企业3户，农产品加工产值增幅超过20%。完善产业化利益分配机制，新增农民专业合作社100家以上。提高规模种植、养殖比例，引导土地承包经营权规范有序流转。加快发展生态水产、特色畜禽、精细蔬果、苗木花卉等优势特色农业，加强名特优农产品基地建设，办好现代农业博览会。加强绿色生产，从源头上保障农产品质量安全。推进“新网工程”建设，促进农业科技推广与应用，提升农业机械化水平。

深入推进美好乡村建设。重点推进7条示范线48个中心村、12个精品村、60个示范村建设，改善村容村貌。实施新一轮农村电网升级改造，改造提升县乡道路，改建50公里通村路，改造20座危桥。开展村庄环境连片整治，完善垃圾污水处理设施，推进农村危房改造工程。加强滁河及中小河流治理，推进农田水利基本建设，实施32座小型水库除险加固工程。加大农村社会事业投入，提高农村基本公共服务水平。强化农民就业技能培训，造就更多适应市场经济需要的新型农民。

创新农村经营体制机制。构建农村综合产权交易平台，实现农村集体资产资源保值增值。鼓励农户采用土地承包经营权入股方式，组建土地股份合作社，参与专业合作社的生产经营，探索开展土地托管和土地预流转等工作。深化林权制度配套改革。继续推进扩权强镇试点，完善中心镇管理职能。探索户籍制度改革，有序推进农业转移人口市民化。

（四）着力完善适应科学发展的体制机制

深化行政管理体制改革。探索新一轮政府机构改革，开展“大部制”改革试点，转变政府职能，优化运行机制，提高行政效能。进一步简政放权，明确权责关系，健全监督机制。巩固扩大“营改增”试点效果。推进预算编制与管理改革，实行开门办预算，推行政府花钱买服务。强化增收节支，推进财政资金绩效管理。开展公务用车专项治理，稳妥推进公车改革。探索旅游管理体制改革，激发旅游业发展活力。

深化经济管理体制改革。按照“精简、统一、效能”和“小机构、大服务”的原则，创新园区、新区体制机制，探索园区、新区“企业化”管理模式。深化土地收储和投融资管理体制改革，最大限度地整合资源，创新融资产品，降低融资成本。引进和发展各类金融机构，完善支持实体经济发展的地方金融体系。健全信用担保体系，规范发展小额贷款公司，积极防范化解金融风险。推进国有资产管理体制改革，构建国有资产统筹运营体系。做好企业一套表改革、第三次全国经济普查工作。

深化其他各项改革。平稳推动事业单位分类改革，推进执法机构综合设置，加强部门所属事业单位整合。深化事业单位人事分配制度改革，实现由编制管理向人员总量控制过渡，人事管理由固定用人向合同用人、身份管理向岗位管理转变。稳步推进市政、园林、公路养护和市容环卫等城市公用事业改革，实行重心下移、管养分离。巩固提升公立医院改革试点和基层医改成果，深入推进县级公立医院改革。推进住房保障制度改革，健全保障性住房公平分配和运行管理机制，推广廉租房和公租房统筹并轨、租补分离模式。

（五）着力提升承接产业转移和对外开放水平

加快开发园区转型升级。完善各类开发园区规划，强化公共服务、吸纳就业、人口集聚功能，促进开发园区向产城一体、宜居宜业的多功能综合性新区转变。按照分工科学、产业集聚、错位互补、共同发展的要求，优化产业结构，提高产业集中度，国家级开发区重点培育2—3个主导产业，省级开发区重点培育1—2个主导产业。坚持依法依规、集约用地，加快标准化厂房建设，积极争创国家级循环经济示范园区。支持县区所属园区立足自身优势，做大做强优势主导产业，形成特色鲜明、优势互补、活力迸发、竞相发展的县域经济发展格局。

创新优化招商合作方式。加大宣传推介力度，着力招大引强。用足协会、商会、学会、展会资源，进一步提高招商效率。依托主导产业、骨干企业、重点项目，引进上下游产业和企业，拓展延伸产业链，促进产业集群发展。进一步完善驻外招商体制机制，加大资源整合力度，充实招商力量，提高谈判水平，实行招商项目合同管理，突出主导产业和重大项目考评，确保实际利用内资增长20%以上、外资增长10%以上。扶持壮大外贸企业队伍，调整出口结构，开拓新兴市场，中小企业进出口增长20%以上；加强对外交流，鼓励企业“走出去”。扩大宁马、马芜、马宿合作成果。

（六）着力增强城市承载服务功能

加强基础设施建设。围绕建设区域中心城市，结合区划调整，启动城市总体规划修编，引领城市大建设、大发展。全年实施城乡建设项目800个以上，完成投资500亿元。按照适度超前、扩大优势的要求，加强交通基础设施建设，推动长江公路大桥年内早日竣工通车，推进城际铁路建设，协调推动苏341省道开工建设，加快博宁通道、宁丹公路前期工作。建设“江西”组团互联大动脉，马巢高速建成通车，加快马滁高速建设和206、226省道改造。实现“江东”组团无缝对接，314省道升级改造全面完工，完成205国道南段改造，推进江东大道南延工程，开展313省道改造前期工作。统一规划、统一管理、科学利用港口岸线资源，用足用好用活黄金水道。完善提升中心城区路网，实施江东大道、慈湖河路综合改造，打通城区“断头路”，畅通城市“微循环”，缓解局部节点交通拥堵状况。加快城际铁路站前广场配套工程、综合客运枢纽站、政务服务中心建设，完成城市出入口环境整治，实施旧城改造。数字化城市管理平台建成运营，严格城市管理考核，加强小区物业管理。

推进生态文明建设。深入开展森林增长工程美化家园活动，实施雨山河、江东大道沿线防护林带建设，启动城市绿道工程，开工建设秀山城市森林花源，实施矿山生态森林植被恢复工程，完成造林绿化14万亩。加快慈湖河中游生态环境综合整治步伐，建成滨江文化湿地公园。大力推进节能减排，加强重点节能工程建设，提升资源再利用水平；城市生活垃圾发电项目开工，启动老旧小区雨污分流工程建设，完善污水处理厂外部管网配套。严格落实节能减排目标责任制，切实解决好群众关心的突出环境问题。

（七）着力加强社会建设和创新社会管理

推动文化繁荣发展。推进社会主义核心价值体系建设，实施公民道德建设工程，巩固提升全国文明城市创建水平。加快发展文化事业，确保国家公共文化服务体系示范区创建通过达标验收，改造市博物馆，规划文化广电中心项目，办好李白诗歌节。大力发展文化产业，加快文化产业园区建设。深入推进全民健身计划，积极备战省十三运会，市综合体育馆、会展中心投入运行。

切实保障和改善民生。坚持民生优先，实施40项民生工程，投入资金36.5亿元，增长21%。鼓励和扶持创业，帮助困难群体就业，实施农村富余劳动力转移就业工程，推进城乡统筹就业，办好人力资源服务产业园，确保城镇新增就业4万人。加强职业技能培训，增强就业稳定性。统筹推进城乡社会保障体系建设，建立城乡居民大病补充医疗保险制度，完善被征地农民基本养老保障政策。发展社会福利、社会救助、社会优抚和慈善事业，更多地帮助关爱残疾人。重视并大力发展老龄事业，健全政府主导、多方参与的社会养老服务体系，努力实现老有所依、老有所养、老有所乐、老有所学、老有所为的目标。推进保障性安居工程建设，加大安置房建设力度。继续实施公交优先发展战略，延伸拓展公交线路，加快发展绿色公交。

统筹发展各项社会事业。全面完成学前教育三年行动计划，推动义务教育均衡发展，切实保障农民工子女、留守儿童和特殊群体平等接受教育的权利，增强职业教育社会服务能力，支持高等教育加快发展。努力提高医疗服务水平，加强医院改革管理，引进优质医疗资源，建设区域卫生信息化平台，建立完善市级医院与基层社区分工协作和双向转诊机制，加快秀山医院建设。稳定低生育水平，综合治理出生人口性别比偏高问题，提高人口素质。推动其他各项社会事业全面进步。支持工会、共青团、妇联等人民团体充分发挥桥梁纽带作用。

促进社会和谐稳定。加强社区建设，推动社区管理体制改革，理顺社会管理组织机构关系，推行扁平化管理。进一步畅通信访渠道，完善三级平台建设，有效化解各类矛盾纠纷。强化安全生产监管，实施食品药品安全放心工程。加强社会治安综合治理，推进科技强警，完善社会治安防控体系，依法打击违法犯罪活动。加强对特殊人群的管理，做好社区矫正和安置帮教工作。完善各类应急预案和处置流程，提高防灾减灾和应对突发事件能力。维护民族团结和宗教和谐稳定。规范市场价格秩序，强化民生价格监管。加强国防教育、国防动员、民兵预备役和人民防空工作，巩固发展军政军民团结。

（八）着力加强政府自身建设

保持更加奋发有为的精神状态。进一步解放思想，更新观念，切实增强市场意识、危机意识和忧患意识，让思想成为行动的先导。强化工作责任心，把工作标准调到最高，把精神状态调到最佳，把工作干劲调到最大，做到我的岗位我负责、我的工作请放心。心无旁骛，集中精力，满怀激情，把工作当事业干，一件一件地抓，一项一项地做，一个环节一个环节地盯。保持敢作为、勇担当的政治品格，知难而上，排难而进，以对事业、对人民高度负责的精神推进工作。敢抓敢管，认真较真，不做老好人，努力创造经得起组织、群众和历史检验的业绩。

提高依法行政科学施政的能力。坚决贯彻落实市委各项决策部署，自觉接受市人大法律监督、工作监督和市政协民主监督，认真听取各民主党派、工商联、无党派人士和人民团体的意见建议，主动接受社会公众和新闻舆论监督，提高政府决策科学化、民主化、法治化水平，推进依法行政。加强对工作的系统思考和研究，想明白了再说，说了就要做，做的要比说的好。进一步明确责任、分清责任、分级负责，做到事有专管之人、人有明确之责、责有限定之期，努力取得事半功倍的效果。

发扬为民务实清廉的工作作风。密切联系群众，把群众当亲人看，带着深厚感情工作，讲实话、干实事，言必信、行必果。深入基层、深入群众、深入实际，改进调查研究方式方法，多到困难和矛盾集中、群众意见多的地方去，注重解决实际问题。改进会风文风，严格清理、切实减少会议活动，开短会、讲短话，减少文件简报。改善绩效管理和评估办法，强化政务督查考核，真干实干说话，数据实绩说话，形成鼓励干事创业的浓厚氛围。加强廉政教育和制度建设，全面推进廉政风险防控，加强审计监督，确保廉洁从政各项规定落到实处。厉行勤俭节约，反对奢侈浪费，压缩行政经费，把有限的财力用于发展经济、改善民生。

各位代表！任务艰巨而繁重，责任重大而光荣。让我们在省委、省政府和市委的坚强领导下，紧紧团结和依靠全市人民，进一步解放思想，振奋精神，鼓足干劲，凝聚力量，为建成更高水平小康社会、在全省率先实现现代化、加快建设区域中心城市而努力奋斗！
